# Supplementary material for: The Andean Ibis (Theristicus branickii) in South America: potential distribution, presence in protected areas and anthropic threats
Source: PeerJ. 2023 Dec 11;11:e16533. doi: 10.7717/peerj.16533 (PMC10720468; doi:10.7717/peerj.16533)
Supplement: Supplemental Information 1 [file peerj-11-16533-s001.docx]

S1 Table:

Habitat factor requirements of the Andean Ibis in South America, based on the literature review.

| **Variable** | **Justification** | **Study reference** |
| --- | --- | --- |
| Altitude:  Slope: | Restricted presence limited to  3700-4500 m a s l.  <20% higher detection of the species probability | BirdLife International. 2017. *Theristicus branickii* (amended version of 2016 assessment). IUCN Red List Threat. Species 2017e.T22697444A112401898. Available at www.iucnredlist.org [Accessed 3 January 2019].  Naveda-Rodríguez, A., T. Santander, V. Utreras B., & G. Zapata-Ríos. 2020. Estimating the Abundance of Andean Ibis (*Theristicus branickii*) in the High Andes of Northern Ecuador. Trop. Conserv. Sci. 13: 1940082920903185. Available at https://doi.org/10.1177/1940082920903185. |
| Roughness  Index | From field observations studies, nest and roosting sites are located in the cliffs  From 1 to 7 and 5 to 7 | Alcocer R. 2014. Nidificación, vigilancia del nido y éxito reproductivo de la bandurria andina *Theristicus melanopis branickii* Berlepsh & Stolzmann, 1894 (Threskiornithidae), en la localidad de Collpamayo, provincia de Espinar, Cusco 2013-2014. Honor tesis. Universidad Nacional de San Agustin de Arequipa. Peru.  Luzuriaga-Neira N, Cuichán D, da Costa M, Reyes N. 2021. Observations on the breeding biology of the Andean Ibis (Theristicus branickii) in Ecuador. *Ornithology Research*. DOI: 10.1007/s43388-021-00044-3.  Naveda-Rodríguez, A., T. Santander, V. Utreras B., & G. Zapata-Ríos. 2020. Estimating the Abundance of Andean Ibis (*Theristicus branickii*) in the High Andes of Northern Ecuador. Trop. Conserv. Sci. 13: 1940082920903185. Available at https://doi.org/10.1177/1940082920903185 |
| Normalized Difference Vegetation Index | Paramo, Puna, Open grassland habitats near water outcrops, rocky areas, used for roosting and nesting; can tolerate agricultural areas in wetlands. | Schulenberg, T. S.; Stotz, D. F.; Lane, D. F.; O'Neill, J. P.; Parker III, T. A. 2007. Birds of Peru. Princeton University Press, Princeton, NJ, USA.  del Hoyo, J., N. Collar, & E. Garcia. 2020. Andean Ibis (Theristicus branickii). *in* Birds of the World.  Luzuriaga N, Cuichan D, Catagña D, Da Costa M, Reyes N. 2019. Description of the ecological reproductive patterns and survival rates of the Black-faced Ibis (*Theristicus branickii* ) in the Antisana Ecological Reserve- Ecuador. In: XI Congreso de Ornitología Neotropical. Costa Rica, 1.  Luzuriaga-Neira, N., D. Cuichán, M. da Costa, & N. Reyes. 2021. Observations on the breeding biology of the Andean Ibis (*Theristicus branickii*) in Ecuador. Ornithol. Res. Available at <https://doi.org/10.1007/s43388-021-00044-3>. |
| Bio 1- Bio 19 | Important bioclimatic factors of the Andean Ibis habitat, based on other avian species from Andean highlands | Naveda-Rodríguez, A., T. Santander, V. Utreras B., & G. Zapata-Ríos. 2020. Estimating the Abundance of Andean Ibis (*Theristicus branickii*) in the High Andes of Northern Ecuador. Trop. Conserv. Sci. 13: 1940082920903185. Available at <https://doi.org/10.1177/1940082920903185>.  Padrón, R. S., B. P. Wilcox, P. Crespo, & R. Célleri. 2015. Rainfall in the Andean páramo: New insights from high-resolution monitoring in southern Ecuador. J. Hydrometeorol. 16: 985–996. |
|  |  | Buytaert, W., V. Iñiguez, & B. De Bièvre. 2007. The effects of afforestation and cultivation on water yield in the Andean paramo. For. Ecol. Manage. 251: 22–30. Available at [http://www.sciencedirect.com/science/article/pii/S0378112707004641.](http://www.sciencedirect.com/science/article/pii/S0378112707004641) |
|  |  | Ministerio del Ambiente del Ecuador. 2013. Mapa de Bioclima. Quito, Ecuador: Ministerio del Ambiente.  Padrón, R. S., B. P. Wilcox, P. Crespo, & R. Célleri. 2015. Rainfall in the Andean páramo: New insights from high-resolution monitoring in southern Ecuador. J. Hydrometeorol. 16: 985–996. |
| River and lakes distance | Based on Field observations and local studies: species was registered near to water bodies | Vizcarra, J. 2009. Observaciones de *Theristicus melanopis melanopis* y *Theristicus melanopis branickii* en el distrito de Ite, sur de Perú. Boletín Chil. Ornitol. 15: 104.  West B. 2014.New Observations of the Andean Ibis (*Theristicus branickii*, Threskiornithidae): Distribution, Movements, and Behavior Near Volcán Antisana". *Available at* *https://digitalcollections.sit.edu/isp_collection/2019*  Alcocer, R. 2014. Nidificación, vigilancia del nido y éxito reproductivo de la bandurria andina *T~~t~~heristicus melanopis branickii* berlepsh & stolzmann, 1894 (threskiornithidae), en la localidad de C~~c~~ollpamayo, provincia.  Muñoz I, Luzuriaga-Neira N, Heredia A, Gonzalez L, Velastegui J, Olmedo I, Garcìa J, Bonaccorso E. 2021. New records of Andean Ibis Theristicus branickii in Ecuador. Cotinga 43:96–98. |

S2 Table:

All variables proposed for the niche model of the Andean Ibis in South America.

| Variables | Source | Description | Type |
| --- | --- | --- | --- |
| BIO1 | Worldclim: <https://www.worldclim.org/> | Annual mean temperature | Continuous |
| BIO2 |  | Mean diurnal range | Continuous |
| BIO3 |  | Isothermality | Continuous |
| BIO4 |  | Temperature seasonality | Continuous |
| BIO5 |  | Max temperature of warmest month | Continuous |
| BIO6 |  | Min temperature of coldest month | Continuous |
| BIO7 |  | Temperature annual range | Continuous |
| BIO8 |  | Mean temperature of wettest quarter | Continuous |
| BIO9 |  | Mean temperature of driest quarter | Continuous |
| BIO10 |  | Mean temperature of warmest quarter | Continuous |
| BIO11 |  | Mean temperature of coldest quarter | Continuous |
| BIO12 |  | Annual precipitation | Continuous |
| BIO13 |  | Precipitation of wettest month | Continuous |
| BIO14 |  | Precipitation of driest month | Continuous |
| BIO15 |  | Precipitation seasonality (Coefficient of variation) | Continuous |
| BIO16 |  | Precipitation of wettest quarter | Continuous |
| BIO17 |  | Precipitation of driest quarter | Continuous |
| BIO18 |  | Precipitation of warmest quarter | Continuous |
| BIO19 |  | Precipitation of coldest quarter | Continuous |
| Roughness index | Global Multi-resolution Terrain Elevation Data 2010GMTED2010: <http://srtm.csi.cgiar.org> | Topography heterogeneity estimated from GMTE | Continuous |
| Slope |  | Degree | Continuous |
| Elevation |  | m a.s.l. | Continuous |
| Distance to lakes  Presence of rivers | Messager, M.L., Lehner, B., Grill, G., Nedeva, I., Schmitt, O. (2016). Estimating the volume and age of water stored in global lakes using a geo-statistical approach. Nature Communications, 7: 13603. <https://doi.org/10.1038/ncomms13603>  <https://www.hydrosheds.org/products/hydrolakes> | Lake_type  1: Lake  2: Reservoir 3:  Lake control (i.e., natural lake with regulation structure)  And others. | Continuous |
| Vegetation Index  NDVI | https://modis.gsfc.nasa.gov/data/dataprod/mod13.php | NDVI/year | Continuous |


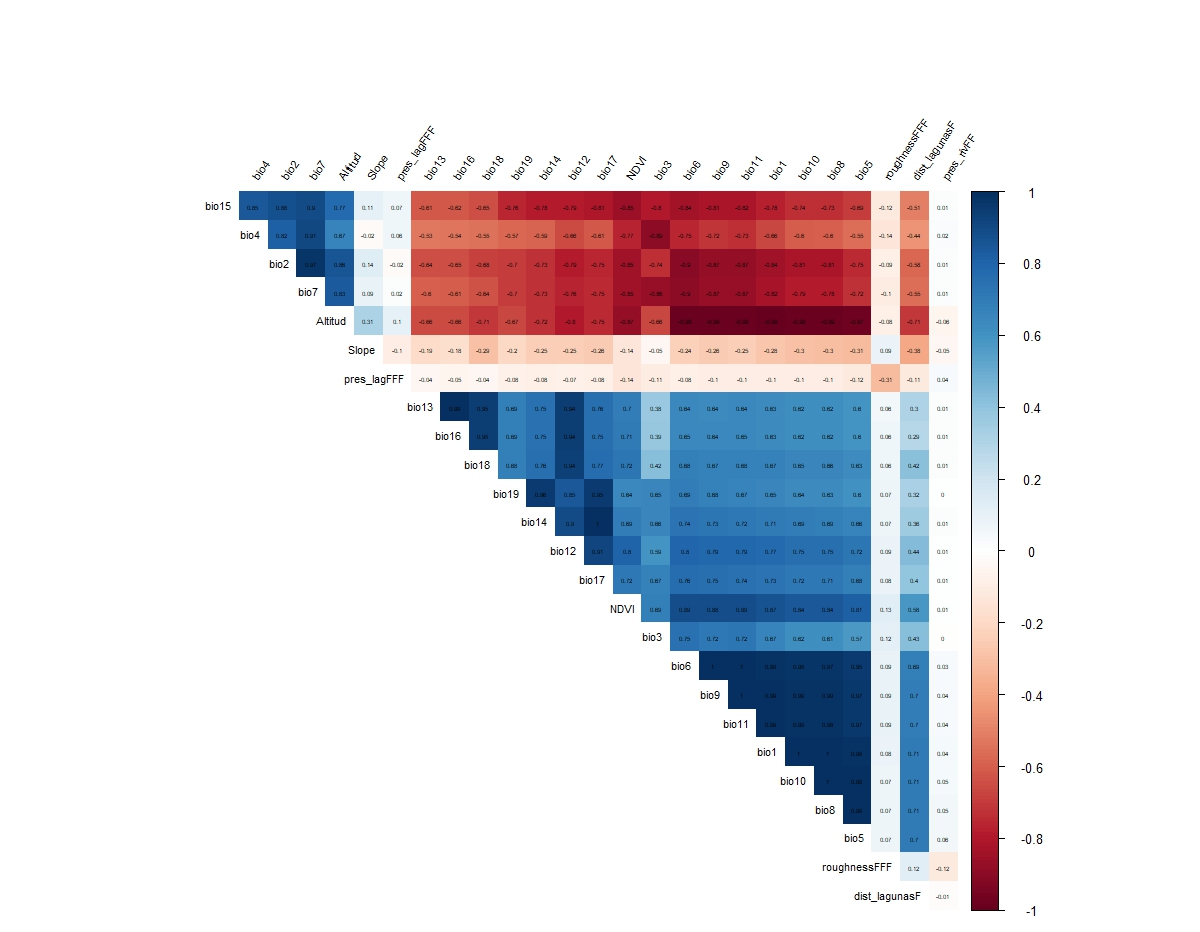


S1 Figure:

Correlation matrix of the proposed 25 bioclimatic and environmental variables to determine the suitable distribution area of the Andean Ibis in South America

Notes: Altitud:Altitude; Slope: Slope; pres_rivFF: Presence of rivers; roughnessFFF: Roughness Index; dist_lagunasF: distance to lagoon; NDVI: Normalized Difference Vegetation Index; bio1: Annual Mean Temperature; bio2: Mean Diurnal Range; bio3: Isothermality; bio4: Temperature Seasonality; bio5: Max Temperature of Warmest Month; bio6: Min Temperature of Coldest Month; bio7: Temperature Annual Range; bio8: Mean Temperature of Wettest Quarter; bio9: Mean Temperature of Driest Quarter; BIO10: Mean Temperature of Warmest Quarter; BIO11: Mean Temperature of Coldest Quarter; BIO12: Annual Precipitation; BIO13: Precipitation of Wettest Month; BIO14: Precipitation of Driest Month; BIO15: Precipitation Seasonality; BIO16: Precipitation of Wettest Quarter; BIO17: Precipitation of Driest Quarter; BIO18: Precipitation of Warmest Quarter; BIO19: Precipitation of Coldest Quarter.

S2 Figure:

Response curves of the nine variables that determine the probability of distribution of the Andea Ibis: figures from the replicate n°10.


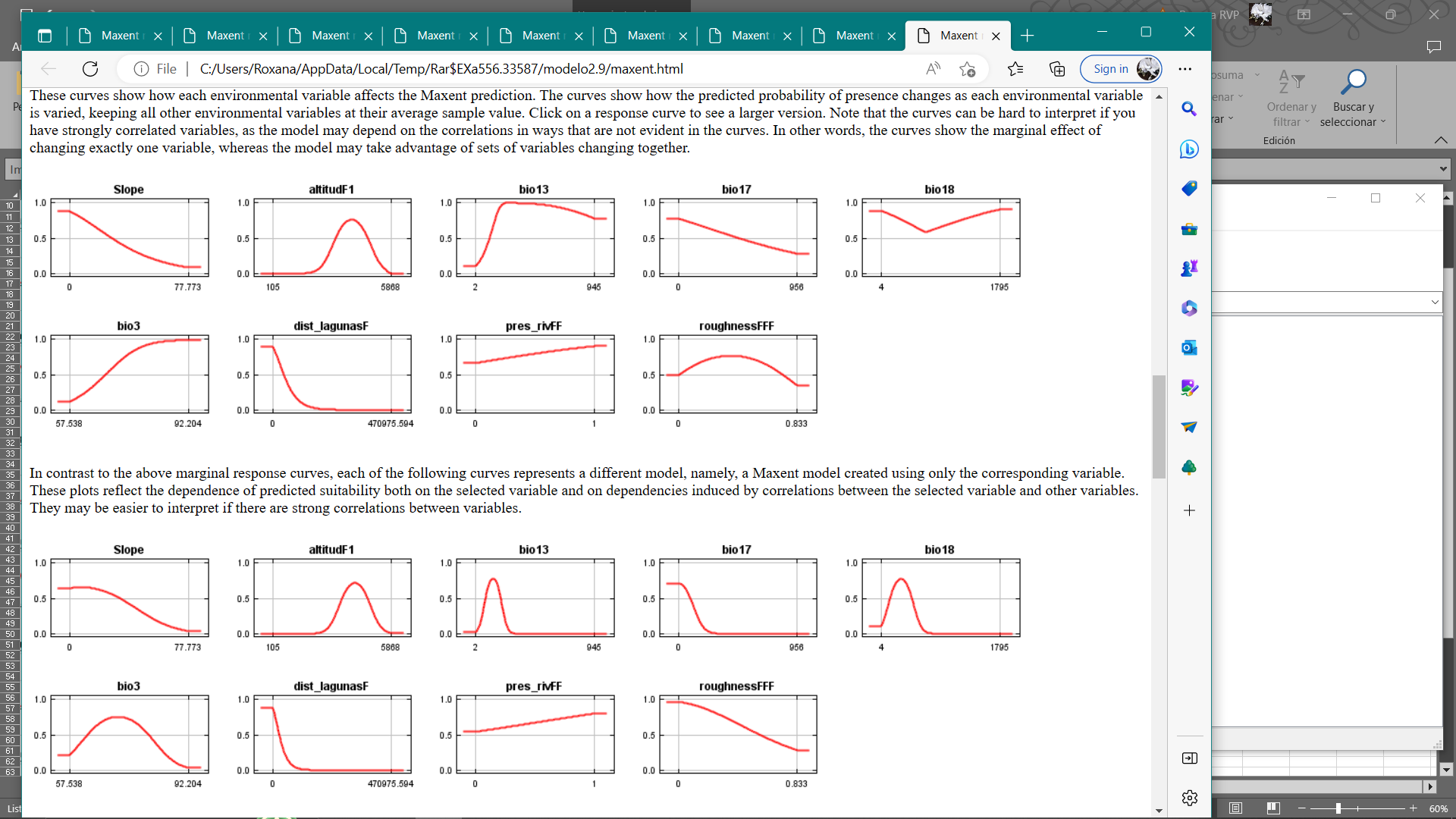


Notes:

Slope: Slope; AltitudF1: Altitude; pres_rivFF: Presence of Rivers; roughnFFF: Roughness Index; dist_lagunasF: distance to lagoons; bio3: Isothermally; bio13: Precipitation of Wettest Month; bio17: Precipitation of Driest Quarter; bio18: Precipitation of Warmest Quarter.

S3 Table:

Presence of Andean Ibis, potential distribution and protected areas in South America. We show each protected area and footprint extension that cover the potential distribution of the species.

|  | **Potential distribution area of the Andean Ibis** | | | | | |
| --- | --- | --- | --- | --- | --- | --- |
| **Country/ Protected area** | **National area (km^2^)** | **National area (%)** | **Footprint (km^2^)** | **Footprint (%)** | **Protected areas (km^2^)** | **Protected areas (%)** |
| **Ecuador** | **21.585,85** | **7,19** | **9.713,12** | 6,82 | **8.443,10** | **28,62** |
| Refugio de Vida Silvestre Pasochoa |  |  | 3,42 |  | 3,52 |  |
| Parque Nacional Cotacachi Cayapas |  |  | 79,77 |  | 306,95 |  |
| Parque Nacional Sumaco Napo-Galeras |  |  | 0,00 |  | 18,52 |  |
| Reserva Ecológica El Ángel |  |  | 39,40 |  | 158,17 |  |
| Parque Nacional Podocarpus |  |  | 1,70 |  | 250,79 |  |
| Parque Nacional Cajas |  |  | 84,15 |  | 295,08 |  |
| Reserva Ecológica Los Ilinizas |  |  | 147,38 |  | 314,12 |  |
| Parque Nacional Cotopaxi |  |  | 174,67 |  | 314,26 |  |
| Reserva de Producción de Fauna Chimborazo |  |  | 418,11 |  | 505,73 |  |
| Reserva Ecológica Antisana |  |  | 31,59 |  | 746,80 |  |
| Parque Nacional Llanganates |  |  | 178,46 |  | 1.161,34 |  |
| Reserva Ecológica Colonso Chalupas |  |  | 0,04 |  | 177,24 |  |
| Parque Nacional Cayambe Coca |  |  | 227,11 |  | 1.570,37 |  |
| Parque Nacional Sangay |  |  | 127,67 |  | 263,69 |  |
| El Boliche |  |  | 3,81 |  | 3,96 |  |
| Área Nacional de Recreación Quimsacocha |  |  | 7,61 |  | 32,32 |  |
| Reserva geobotánica Pululahua |  |  | 0,05 |  | 0,07 |  |
| Parque Nacional Yacuri |  |  | 9,74 |  | 320,17 |  |
| **Perú** | **216.031,33** | **71,99** | **101.801,87** | 71,46 | **13.642,40** | **46,25** |
| Santuario Nacional Megantoni |  |  | 0 |  | 3,53 |  |
| Reserva Nacional Calipuy |  |  | 0,06 |  | 51,22 |  |
| Reserva Nacional Pampa Galeras Barbara D´ Achille |  |  | 10,1 |  | 13,67 |  |
| Parque Nacional Yanachaga-Chemillén |  |  | 0,09 |  | 30,55 |  |
| Santuario Histórico Chacamarca |  |  | 19,46 |  | 25,00 |  |
| Santuario Nacional Ampay |  |  | 19,77 |  | 32,07 |  |
| Santuario Nacional Calipuy |  |  | 10,98 |  | 42,47 |  |
| Santuario Nacional Tabaconas Namballe |  |  | 0,05 |  | 47,94 |  |
| Parque Nacional del Manu |  |  | 6,6 |  | 55,46 |  |
| Santuario Nacional Huayllay |  |  | 54,88 |  | 68,15 |  |
| Coto de caza Sunchubamba |  |  | 80,69 |  | 111,28 |  |
| Santuario Histórico MachuPicchu |  |  | 56,57 |  | 242,83 |  |
| Reserva Nacional de Titicaca |  |  | 18,86 |  | 364,37 |  |
| Bosque de Protección Pui Pui |  |  | 0,09 |  | 452,51 |  |
| Reserva Nacional Junín |  |  | 218,51 |  | 525,50 |  |
| Zona Reservada Cordillera Huayhuash |  |  | 93,08 |  | 652,90 |  |
| Santuario Histórico de la Pampa de Ayacucho |  |  | 0,38 |  | 1,16 |  |
| Parque Nacional Río Abiseo |  |  | 12,11 |  | 708,13 |  |
| Reserva Nacional Salinas y Aguada Blanca |  |  | 287,87 |  | 1.728,07 |  |
| Reserva Paisajística Nor Yauyos – Cochas |  |  | 594,19 |  | 2.203,21 |  |
| Reserva Paisajistica Sub Cuenca del Cotahuasi |  |  | 589,57 |  | 3.036,92 |  |
| Parque Nacional Huascarán |  |  | 711,24 |  | 3.245,46 |  |
| **Bolivia** | **57.020,65** | **19,00** | **30.563,63** | **21,45** | **5.540,40** | **18,78** |
| Parque Nacional Cotapata |  |  | 22,10 |  | 28,60 |  |
| Parque Nacional Madidi |  |  | 37,14 |  | 464,10 |  |
| Parque Nacional Sajama |  |  | 185,15 |  | 485,84 |  |
| Parque Nacional Carrasco |  |  | 113,80 |  | 549,32 |  |
| Parque Nacional Tunari |  |  | 1.591,68 |  | 1.669,88 |  |
| Area Natural de Manejo Apolobamba |  |  | 1.132,58 |  | 2.341,66 |  |
| Parque Nacional Toro Toro |  |  | 0,85 |  | 1,00 |  |
| **Chile** | **5.457,17** | **1,82** | **377,18** | **0,26** | **1.872,00** | **6,35** |
| Parque Nacional Lauca |  |  | 35,77 |  | 286,75 |  |
| Reserva Nacional Las Vicuńas |  |  | 9,94 |  | 1.070,32 |  |
| Monumento Natural Salar de Surire |  |  | 0,09 |  | 61,85 |  |
| Parque Nacional Volcan Isluga |  |  | 17,34 |  | 211,69 |  |
| Salar De Huasco |  |  | 3,93 |  | 125,49 |  |
| Parque Nacional Conguillio |  |  | 0,00 |  | 11,00 |  |
| Parque Nacional Laguna Laja |  |  | 0,00 |  | 6,80 |  |
| Parque Nacional Llullaillaco |  |  | 0,01 |  | 3,83 |  |
| Parque Nacional Radal Siete Tazas |  |  | 0,00 |  | 0,2 |  |
| Parque Nacional Villarica |  |  | 3,84 |  | 7,14 |  |
| Reserva Nacional Altos de Lircay |  |  | 0,00 |  | 0,96 |  |
| Reserva Nacional Malalcahuello |  |  | 0,00 |  | 0,72 |  |
| Reserva Nacional Nalcas |  |  | 0,00 |  | 0,68 |  |
| Reserva Nacional Nuble |  |  | 0,00 |  | 59,44 |  |
| Reserva Nacional Ralco |  |  | 0,00 |  | 10 |  |
| Reserva Nacional Río Cipreces |  |  | 0,07 |  | 13,67 |  |
| Reserva Nacional Río Clarillo |  |  | 0,00 |  | 0,46 |  |
| Reserva Nacional Río Villarica |  |  | 0,00 |  | 1 |  |
| **TOTAL** | **300.095,00** | **100,00** | **142.455,80** | **100,00** | **29.497,90** | **100,00** |
